# Deficient inhibition of irrelevant visual information in migraine: A magnetoencephalography study of alpha‐band activity

**DOI:** 10.1111/head.70108

**Published:** 2026-06-15

**Authors:** Rémy Masson, Alma ElShafei, Geneviève Demarquay, Lesly Fornoni, Yohana Lévêque, Anne Caclin, Aurélie Bidet‐Caulet

**Affiliations:** ^1^ Université Claude Bernard Lyon 1, INSERM, CNRS, Centre de Recherche en Neurosciences de Lyon CRNL U1028 UMR 5292 Bron France; ^2^ Neurological Hospital Pierre Wertheimer, Hospices Civils de Lyon Université Claude Bernard Lyon 1, Université de Lyon Lyon France; ^3^ Aix Marseille Univ Inserm, INS, Inst Neurosci Syst Marseille France

**Keywords:** alpha rhythm, attention, interictal hypersensitivity, magnetoencephalography, migraine without aura

## Abstract

**Objectives/Background:**

There is growing evidence that migraine is associated with attentional abnormalities, both during and between attacks, potentially affecting the cognitive processing of sensory stimulation. However, the underlying neurophysiological mechanism remains poorly understood. This study aimed to investigate whether top‐down and bottom‐up attentional mechanisms, as indexed by alpha‐ and gamma‐band oscillatory activity measured with magnetoencephalography are altered in patients with episodic migraine without aura during the interictal phase.

**Methods:**

In a cross‐sectional study (conducted in 2016–2017 in Lyon area of France) with a comparison of matched groups, 19 patients with migraine without aura and 19 healthy participants performed an attentional task involving visually cued auditory targets and distracting sounds to evaluate conjointly top‐down and bottom‐up attention mechanisms. Magnetoencephalography was used to record anticipatory alpha activity (power increase and decrease) and distractor‐induced gamma activity as markers for top‐down (inhibition and facilitation) and bottom‐up attention, respectively.

**Results:**

Compared to healthy participants, patients with migraine exhibited a significantly less prominent alpha power increase in visual areas in anticipation of the auditory target (permutation tests, cluster significance *p* = 0.043), indicating a reduced inhibition of task‐irrelevant visual areas. In contrast, there was no significant group difference regarding the alpha power decrease in the relevant auditory cortices in anticipation of the target (permutation tests, cluster significance, *p* > 0.310), nor regarding the distractor‐induced gamma power increase in the ventral attention network (permutation tests, cluster significance *p* > 0.999).

**Conclusion:**

These results suggest an impairment of top‐down attentional inhibitory processes in migraine, as reflected by the altered alpha modulation in the visual cortex. This relative inability to suppress irrelevant sensory information may underlie the self‐reported increased distractibility and contribute to sensory disturbances in migraine.

## Plain Language Summary

In addition to the well‐known recurring headache, people with migraine are often overly sensitive to lights, sounds, and smells, even when they do not have a headache. We used brain recordings to study how attention works differently in people with migraine and those without. We showed that patients with migraine have more problems reducing activity in brain visual areas that are not needed when completing a sound‐based task, which might help explain why patients with migraine can be overwhelmed by sensory inputs in real life.

**Abbreviations**
MEG: magnetoencephalography
RON: reorienting negativity
RT: reaction time
rTPJ: right temporo‐parietal junction
VAN: ventral attention network

## INTRODUCTION

Although head pain is undoubtedly the most prominent symptom, migraine is also a disorder of sensory processing.^1^, ^2^, ^3^ Migraine attacks are associated with an aversion to external stimulation across all sensory modalities (photophobia, phonophobia, osmophobia, and allodynia).^4^, ^5^, ^6^, ^7^, ^8^, ^9^ This hypersensitivity often persists in the attack‐free period, albeit to a lesser extent.^8^, ^10^, ^11^ The most prominent model to explain these sensory disturbances, particularly during the interictal state, is a deficit of habituation to repeated sensory stimulation.^12^, ^13^ However, over the last decade, converging pieces of evidence have suggested that dysfunctional attention processing of sensory inputs may also contribute to migraine hypersensitivity. People with migraine report attentional difficulties in everyday life,^14^, ^15^ and these complaints correlate with multimodal sensory sensibility between attacks.^14^ Electrophysiological studies have shown heightened markers of attention orienting to incoming stimuli,^16^, ^17^, ^18^, ^19^ which could be related to the dysfunction of the right temporo‐parietal junction (rTPJ).^18^, ^20^, ^21^, ^22^ Abnormal attention orienting has also been proposed to participate in the sensory discomfort of other disorders such as autism and attention deficit disorders.^23^, ^24^, ^25^, ^26^ Likewise, a deficient attention filter in migraine may lead to an abnormal management of sensory inputs, exposing patients to a state of hyperresponsiveness.

The brain relies on a balance between top‐down and bottom‐up attentional processes to select relevant information in an environment rich in sensory input. Top‐down attention is voluntary and goal‐oriented; it promotes the processing of task‐relevant information through facilitatory mechanisms and suppresses the processing of irrelevant information through inhibitory mechanisms.^27^, ^28^, ^29^, ^30^ In contrast, bottom‐up attention is involuntary and stimulus‐driven; its role is to maintain responsiveness to unexpected but meaningful events through automatic attention shifts that override goal‐directed processes.^31^, ^32^, ^33^

Alpha rhythms (7–15 Hz) are considered to reflect functional inhibition in task‐relevant and irrelevant cortical areas,^34^, ^35^ with increases and decreases in alpha power possibly reflecting inhibitory and facilitatory top‐down processes, respectively.^36^, ^37^, ^38^ In humans, the causal role of alpha in modulating attention has been demonstrated using a neurofeedback procedure.^39^ On the other hand, gamma‐band activity (>30 Hz) would reflect the activation of the cortical areas from which they are generated^40^ and has been consistently associated with feedforward pathways directing information from primary sensory areas to associative areas.^41^, ^42^ Thus, gamma rhythms have been proposed to participate in bottom‐up attentional processes.^39^, ^43^ However, this dichotomy may oversimplify a more complex reality; gamma activity has also been associated with top‐down attention.^44^, ^45^, ^46^

Brain rhythms have been investigated in migraine for decades, providing inconsistent results, except for the recurrent observation of visual cortices hyperexcitability in this population.^47^ Magnetoencephalography (MEG) is a method of choice for the noninvasive investigation of brain activity in the frequency domain.^43^, ^44^ A recent review^48^ identified only 38 MEG studies in migraine, focusing on either resting‐state data or event‐related fields (mostly during visual processing or finger‐tapping tasks). However, no MEG study to date has investigated frequency‐specific neural dynamics subtending attentional processes in migraine.

In the present study, we analyzed brain activities in the frequency domain using MEG during an attention task evaluating conjointly top‐down and bottom‐up attention. Taking advantage of previous extensive studies using the same paradigm in healthy participants,^36^, ^43^, ^49^ we measured alpha and gamma power as markers of top‐down and bottom‐up attentional processes, respectively. Based on previous work using other measures of brain activity,^18^, ^50^ we tested alterations for both top‐down and bottom‐up mechanisms in migraine. We hypothesized that, compared to healthy controls, patients with migraine would show (1) reduced top‐down inhibition of task‐irrelevant brain areas (indexed by decreased alpha power in visual areas), (2) reduced top‐down facilitatory processes in task‐relevant areas (indexed by larger alpha power in auditory and motor cortices), and (3) enhanced bottom‐up attention triggered by auditory distractors (indexed by greater gamma power in the ventral attention network [VAN]).

## METHODS

The present study is a cross‐sectional study with a comparison of matched groups. This preplanned analysis of brain activities in the frequency domain is a second analysis of previously collected data.

The first analysis compared event‐related potentials/fields between patients with migraine and healthy control participants and can be found in a previous publication.^18^ The task, procedure, and MEG preprocessing remain identical. Data from 10 control participants are also part of another study.^43^ This study was approved by the Comité de Protection des Personnes Sud‐Est III (number 2014‐050 B) and registered as a clinical trial under the number NCT02791997 (February 6, 2015). Data were collected in 2016–2017, and all participants were recruited in Lyon (France) and surrounding areas.

### Participants

Twenty‐five patients (17 female, eight male) with migraine without aura were included in this study. Inclusion criteria were age between 18 and 60 years and a diagnosis of episodic migraine with a reported migraine frequency between 2 and 5 days per month. Exclusion criteria were migraine with aura, chronic migraine, and current use of migraine‐preventive medication. All patients were examined by a neurologist (G.D.). Because the aim was to assess attentional processing during the interictal state, the testing session was postponed to a later date if a migraine attack occurred within 72 h before the session.^51^ Additionally, data were excluded from the analyses if the patient had a migraine attack during the 72 h after the session.^51^ Data from 19 patients (13 female, six male) were analyzed. Data from five patients were excluded due to a migraine attack occurring within the 72 h post‐recording session, and one additional patient was excluded for task performance issues. All participants with migraine completed the Headache Impact Test 6 and the Migraine Disability Assessment scales to assess the severity of the disease.^52^, ^53^ No statistical power calculation was conducted prior to the study, and the sample size was based on our prior experience with MEG studies using a similar protocol^36^, ^43^ and electroencephalographic (EEG) studies investigating migraine.^16^, ^19^

From a database of control participants, 19 healthy control participants free of migraine and matched (at the group level) to patients for sex, age, handedness, education level, and musical training (expressed as number of years of formal musical training) were selected. Matching was successful, because we did not observe any significant differences between groups on any of these variables (Table 1), which were thus not included as covariates in the main analyses. Musical training was matched between groups because pitch discrimination was required in the task described below and is known to improve with musical practice. Exclusion criteria for all participants included a medical history of psychiatric or neurological disorders (except migraine without aura in the patient group), current medical treatment (other than contraceptive medication), pregnancy, and hearing disability. As migraine is often associated with increased anxiety and depression,^54^, ^55^, ^56^ and all patients and 17 out of 19 control participants completed the Hospital Anxiety and Depression Scale (HADS) questionnaire^57^ to verify that anxiety and/or depression were not confounding variables (for this variable only, group comparisons are thus based on the comparison of data from 19 patients and 17 control participants). The normal distribution of the demographic data was tested using Shapiro–Wilk tests. The distributions of musical training (*W* = 0.795, *p* < 0.001), HADS depression score (*W* = 0.875, *p* = 0.018), and Migraine Disability Assessment score (*W* = 0.866, *p* = 0.013) in patients with migraine, and of age (*W* = 0.878, *p* = 0.019), musical training (*W* = 0.725, *p* < 0.001), and HADS depression score (*W* = 0.811, *p* = 0.003) in controls were not normal. Group differences were tested using nonparametric Mann–Whitney *U* tests. All demographic statistics are reported in Table 1. All participants gave written informed consent and received monetary compensation for their participation.

**TABLE 1:** Demographics and headache profile of the control and migraine groups.

	Migraine	Control	*p*‐value
Sample size	19	19	–
Age, years, median [Q1–Q3]	30.7 [26.4–37.1]	28.3 [25.8–34.0]	0.530
Sex, no. (percentage) of female participants	13 (68%)	13 (68%)	–
Education level, years, median [Q1–Q3]	15.0 [14.5–17.0]	16.0 [14.5–17.0]	0.988
Musical training, years, median [Q1–Q3]	1.0 [0.0–5.5]	0.0 [0.0–4.5]	0.740
Handedness, no. of right‐handed participants	19	19	–
Anxiety score (HADS), for control participants only, *n* = 17, median [Q1–Q3]	5.0 [3.5–7.5]	5.0 [3.0–6.0]	0.416
Depression score (HADS), for control participants only, *n* = 17, median [Q1–Q3]	2.0 [0.5–4.0]	2.0 [0.0–2.0]	0.306
Migraine duration, years, median [Q1–Q3]	14.5 [1.5–22.8]	NA	–
HIT‐6 score, median [Q1–Q3]	63.3 [61.0–67.5]	NA	–
MIDAS score, median [Q1–Q3]	8.0 [5.0–20.2]	NA	–

*Note*: Two control participants did not complete the HADS questionnaire. Sex and handedness were strictly matched between groups. For other variables, median, Q1, and Q3 values are provided and group differences are tested using a nonparametric Mann–Whitney *U* test. Maximum scores for the Anxiety and Depression subscales of the HADS are 21. HIT‐6 scores can be between 36 and 78 (with higher scores for higher impact of headaches). MIDAS scores are the number of days of incapacitation over the last 3 months.

Abbreviations: HADS, Hospital Anxiety and Depression Scale; HIT‐6, Headache Impact Test 6; MIDAS, Migraine Disability Assessment; NA, not applicable; Q1, first quartile; Q3, third quartile.

### Task and procedure

**FIGURE 1:**
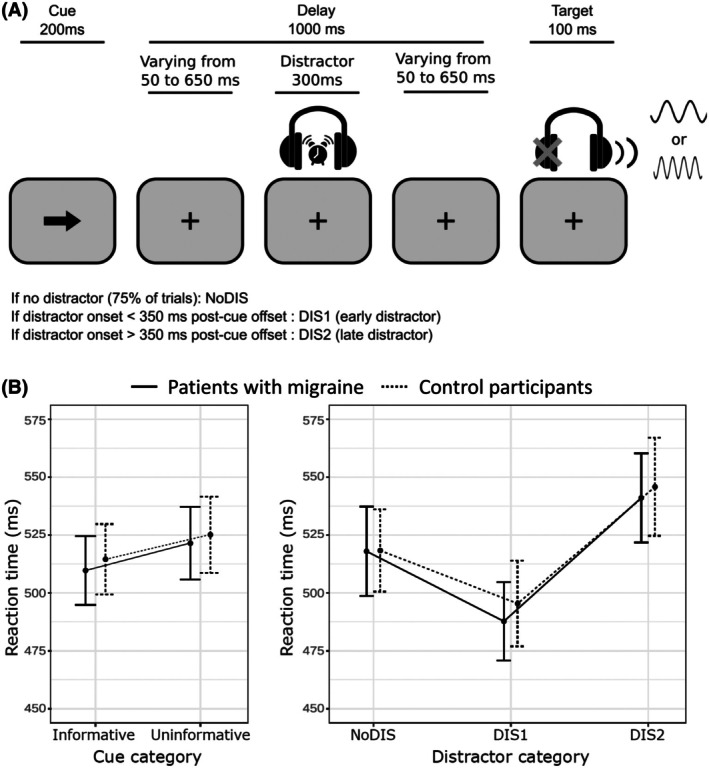
(A) Protocol. The task was to discriminate between a low‐ and a high‐pitched sound, presented monaurally. A visual cue initiated the trial, and was either informative (50%, one‐headed arrow, as shown in the figure) or noninformative (50%, double‐headed arrow, not shown in the figure) about the target ear. Twenty‐five percent of the trials included a distracting sound during the cue target interval at a random delay after the cue offset. The DIS1 condition corresponds to early distracting sounds (starting 50–350 ms after cue offset), and the DIS2 condition corresponds to late distracting sounds (starting 350–650 ms after cue offset). (B) Behavioral results. Mean (across participants) of the median reaction time (median reaction time computed per participant and condition), as a function of the cue and group category on the left panel, and as a function of the distractor and group category on the right panel. Error bars represent the standard error of the mean. See Masson et al.^18^ for more details. Figure adapted from Masson et al.^18^ DIS1, early distractor; DIS2, late distractor; NoDis, no distractor.

Participants performed the Competitive Attention Test (Figure 1),^58^, ^59^, ^60^, ^61^ an audiovisual attention task derived from the Posner Task, designed to produce robust measures of both top‐down and bottom‐up attention. For a detailed description of the protocol used in the present study, please see ElShafei et al.^43^

In short, all trials comprised a visual cue followed 1000 ms later by a monaural auditory target. Half of the time, the cue was a one‐headed arrow and was informative on the side of presentation of the target sound; the other half of the time, the cue was a two‐headed arrow and was not informative on the side of presentation of the target sound. Participants were asked to discriminate between high‐pitched and low‐pitched target sounds and to respond as fast as possible using a joystick.

In 25% of the trials, a salient task‐irrelevant environmental binaural sound was played at some point between the cue offset and the target onset. If the distracting sound onset was early (50 to 350 ms after cue onset), the trial was categorized as DIS1; if the distracting sound onset was late (350 to 650 ms after cue onset), the trial was categorized as DIS2. In the 75% remaining trials, no distracting sound was played; those trials were categorized as NoDIS.

Full description of the analyses performed on behavioral data can be found in Masson et al.^18^ We interpreted the difference in reaction times (RTs) between trials with informative and noninformative visual cues as a proxy of top‐down orienting processes, the difference in RTs between trials with early (DIS1) and late (DIS2) distractors as a proxy of bottom‐up distraction processes, and the difference in RTs between trials without distractors and trials with an early distractor (DIS1) as a proxy of phasic arousal.

### MEG recording and preprocessing

Simultaneous EEG and MEG data were recorded with a sampling rate of 600 Hz during task performance. A 275‐channel whole‐head axial gradiometer system (CTF‐275; VSM Medtech, Vancouver, Canada) was used to record electromagnetic brain activity (0.016–150 Hz filter bandwidth and third‐order spatial gradient noise cancellation). Head movements were continuously monitored using three coils placed at the nasion and the two preauricular points. EEG was recorded continuously from seven scalp electrodes and electrooculogram (EOG) with one bipolar derivation. Please note that EEG data are not presented in this article, because they were only collected for comparison with previous event‐related potential studies (see Masson et al.^18^ for more details).

For each participant, a three‐dimensional magnetic resonance imaging scan was obtained using a 3T Siemens Magnetom whole‐body scanner (Erlangen, Germany); locations of the nasion and the two preauricular points were marked using fiducials markers. These images were used for reconstruction of individual head shapes to create forward models for the source reconstruction procedures.

MEG data were processed offline using the software package for electrophysiological analysis (ELAN Pack) developed at the Lyon Neuroscience Research Center.^58^ Continuous MEG data were bandstop‐filtered between 47 and 53 Hz, 97 and 103 Hz, and 147 and 153 Hz (zero‐phase shift Butterworth filter, order 3) to remove power‐line artifacts. An independent component analysis was performed on the 0.1–40 Hz band‐pass filtered MEG signal to isolate eye movements and heartbeat artifacts. Component topographies and time courses were visually inspected to determine which ones were to be removed through an independent component analysis inverse transformation. There were two to five components removed from the bandstop‐filtered MEG signal in each participant.

Only trials in which the participant answered correctly were retained. Trials contaminated with muscular activity or any other remaining artifacts were excluded automatically using a threshold of 2200 femtotesla for MEG channels. Trials for which the head position differed by >10 mm from the median position during the 10 blocks were also excluded from the analyses. For all participants, >80% of trials remained in the analyses after rejection (corresponding to at least 510 remaining trials per participant, including roughly 380 trials with no distractors and 130 trials with distractors).

In anticipation of the baseline correction for distractor‐related activity in further analyses, for each distractor onset time range, surrogate distractors were created in the NoDIS trials with similar distribution over time than the real distractors.

### Time‐frequency analyses

All further analyses were conducted using the Fieldtrip MATLAB toolbox (MATLAB version 2015a; The MathWorks, Natick, MA; Fieldtrip release version 20151231^59^). Before analysis in the frequency domain, for each event, the time‐locked response (event‐related field) was removed to analyze only induced activity free from any evoked activity.

#### Sensor‐level activity

Time‐frequency power was calculated using Morlet wavelet decomposition with a width of four cycles per wavelet (*m* = 7^60^) at center frequencies between 1 and 150 Hz, in steps of 1 Hz.

For cue‐related alpha activity, baseline correction was performed by computing relative change between activity (low‐alpha: 7–11 Hz; high‐alpha: 11–15 Hz, 0–1800 ms postcue) and baseline activity (−600 to −200 ms precue, averaged over time). For distractor‐related gamma activity, baseline correction was performed by computing relative change between activity (60–100 Hz, 0–350 ms postdistractor) in response to distractors versus surrogate distractors.

#### Source‐level activity

We utilized the frequency domain adaptive spatial technique of dynamical imaging of coherent sources^61^ to reconstruct alpha and gamma activities in the source space dimension. Cross‐spectral density matrices were calculated using the multitaper method from −600 to 2000 ms relative to cue onset (lamda 5%) with a target frequency of 11 (± 4) Hz for NoDis trials, and from −100 to 350 ms relative to distractor onset (lamda 5%) with a target frequency of 80 (±20) Hz for all trials. For each participant, an anatomically realistic single‐shell head model based on the cortical surface was generated from individual head shapes.^62^ A grid with 0.5 cm resolution was normalized on a Montreal Neurological Institute (MNI) template and then morphed into the brain volume of each participant. Lead fields for all grid points along with the cross‐spectral density matrix were used to compute a common spatial filter that was used to estimate the spatial distribution of power for the time‐frequency window of interest.

#### Statistical analysis

Demographic variables were compared between groups with Mann–Whitney *U* tests (Table 1). As previously reported in Masson et al.,^18^ median RTs for correct responses (Figure 1B) were computed for each participant and condition, and were analyzed with repeated‐measures analysis of variance (ANOVA) tests (see results for details on factors). Normality was tested using Shapiro–Wilk tests. Because all *p*‐values were superior to 0.08, we could thus proceed to parametric ANOVA. Greenhouse–Geisser correction was applied to correct for violation of the sphericity assumption.

To visualize source‐level MEG activity for each event, in each participant group, we contrasted baseline activity (either prestimulus activity for cue‐related activities or surrogate distractor‐related activity for the real distractor‐related activity) to the activity of interest, using nonparametric cluster‐based permutations tests that control for multiple comparisons (the cluster‐forming threshold was adapted to each group for visualization purposes when needed to reduce overlap between clusters of activity) in the source space dimension.^63^ The choice of time‐frequency windows of interest was informed by the results from previous studies using this paradigm^36^, ^43^, ^49^ and observation of sensor‐level data. High (11–15 Hz) and low (7–11 Hz) alpha‐band activities were investigated between 700 and 1100 ms post‐cue onset (i.e., in anticipation of the target sound that was played at 1200 ms postcue). Gamma‐band activity (60–100 Hz) was investigated between 100 and 300 ms after the distracting sound onset. Based on previous results,^36^, ^43^ we expected a decrease in low alpha and an increase in high alpha after the cue and an increase in gamma after the distractor. Therefore, we used one‐tailed tests in the nonparametric cluster‐based permutation approach. A complex lateralization pattern of the power decrease in the low‐alpha frequency band was found according to the cue direction in a previous study,^36^ using 33% of each cue category (left, right, uninformative). In the present study, to reduce the experiment's duration, we used 25% of left, 25% of right, and 50% of uninformative cues, precluding such lateralization analysis.

To compare brain activity between controls and particpants with migrain, at the sensor and source levels, we first baseline‐corrected the activities (subtraction of baseline activity from the activity of interest) and tested the group effect on the resulting baseline‐corrected activities using nonparametric cluster‐based permutations tests (with a cluster‐forming threshold of 0.05). Because we could not predict the direction of the effects, we used two‐tailed tests for comparing groups.

## RESULTS

Twenty‐five patients with migraine without aura were included in this study. Data from five patients were excluded due to a migraine attack occurring within the 72 h post‐recording session, and one additional patient was excluded for task performance issues. Data from 19 patients (13 female, six male) were thus included in the following analyses.

A full description of the behavioral results is available in Masson et al.^18^ In summary, median RTs for correct responses (Figure 1B) were computed for each participant and condition and were analyzed with a three‐way ANOVA with group as a between‐subject factor, and with cue category (two levels: uninformative, informative) and distractor condition (three levels: NoDIS, DIS1, DIS2) as within‐subject factors (Greenhouse–Geisser correction was applied to correct for violation of the sphericity assumption). There was no significant group difference (*F*_1,36_ = 0.013, *p* = 0.909) nor any interaction between group and the other two factors in these ANOVAs (group by cue: *F*_1,36_ = 0.043, *p* = 0.837; group by distractor: *F*_2,72_ = 0.223, *ℇ* = 0.823, *p* = 0.757).

### Cue‐elicited alpha‐band activities

#### Anticipation of the target sound: Alpha‐band activities

**FIGURE 2:**
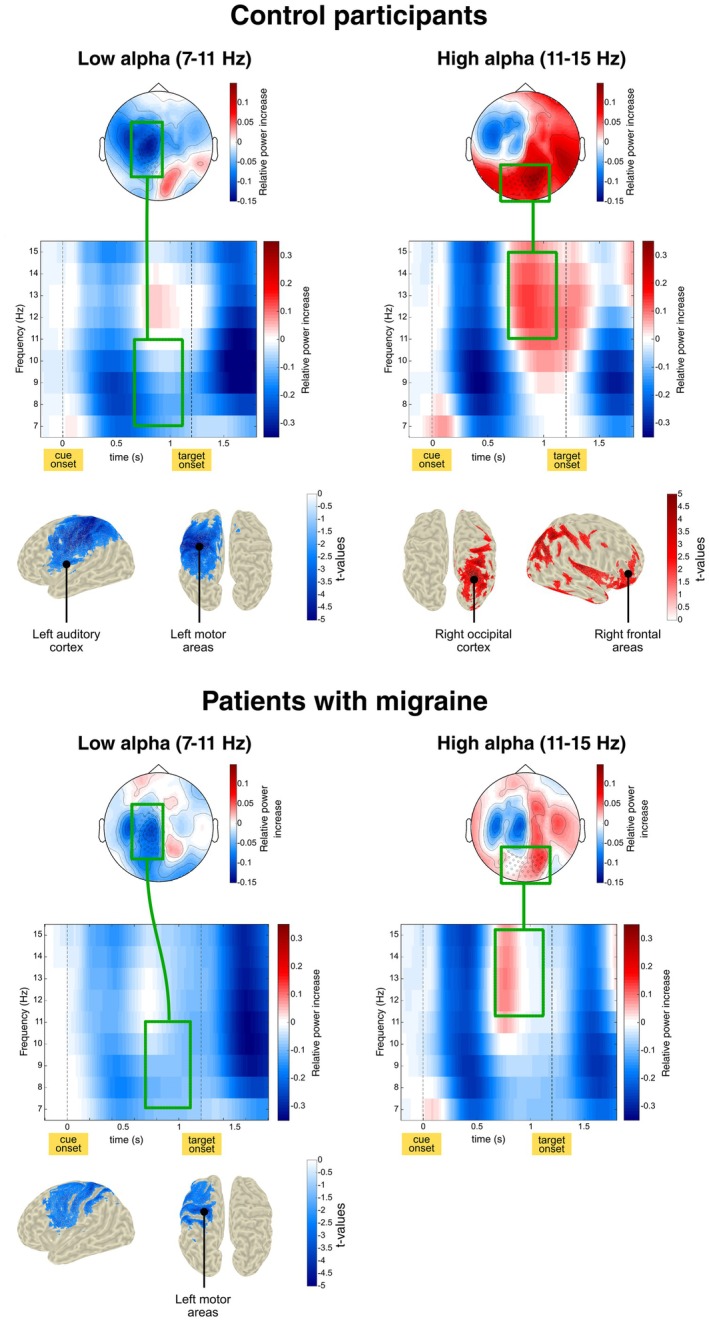
Cue‐related low‐ and high‐alpha activity in each group. For each group, the low‐alpha (7–11 Hz) activity is presented on the left panel, and the high‐alpha (11–15 Hz) is presented on the right panel. (Top) Scalp topographies of the baseline‐corrected low‐ or high‐alpha power during target anticipation (time window of interest: 0.7–1.1 s post‐cue onset; baseline window: −0.6 to −0.2 pre‐cue onset). (Middle) Time‐frequency visualization of baseline‐corrected alpha power measured at the sensors highlighted by black circles in the topographic maps. (Bottom) Distributions of *t* values, masked at corrected *p* < 0.050, from cluster‐based permutation tests (one‐tailed tests, cluster formation threshold at *p* = 0.050) contrasting low‐ or high‐alpha activity during target anticipation against baseline activity at the source level. For the migraine group, no significant high‐alpha activity emerged in the source‐space analysis. [Color figure can be viewed at wileyonlinelibrary.com]

The anticipation of the auditory target led in the control group to two distinct spatio‐temporal patterns for the low‐alpha (7–11 Hz) and high‐alpha frequency (11–15 Hz) bands at the sensor level, whereas a somewhat different pattern was observed in patients with migraine (Figure 2).

At the sensor level, both control and migraine groups exhibited a decrease of low‐alpha power over left central and temporal sensors in anticipation of the target sound. Source analysis indicated that this decrease of low‐alpha power corresponded to a significant cluster including left motor areas and the left auditory cortex in the control group (*p* = 0.018) and mostly left motor areas in the migraine group (*p* = 0.029).

Control participants also presented an increase of high‐alpha power over the occipital and right temporo‐parietal sensors starting from 700 ms post‐cue onset. Source analysis indicated that this increase of high‐alpha power corresponded to a significant cluster located only in the right hemisphere and which extended over occipital and parietal areas, the auditory cortex, and the orbitofrontal cortex (*p* = 0.007). In the migraine group, the increase of high‐alpha power over occipital sensors was notably short‐lived. Source analysis did not confirm the presence of a significant pattern of high‐alpha power increase in the migraine group (*p* = 0.091).

#### Group differences in alpha‐band activities

**FIGURE 3:**
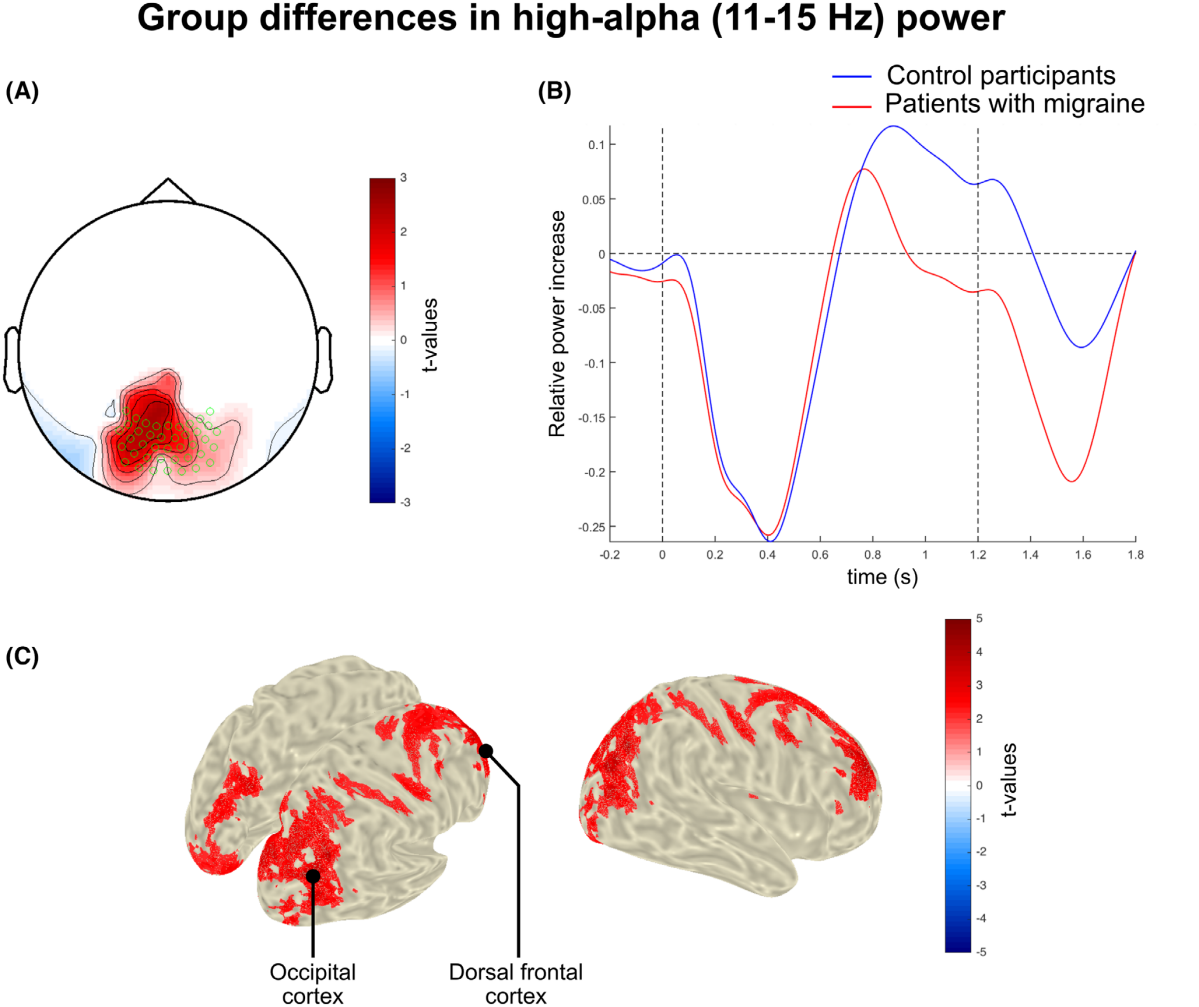
Group differences in cue‐related high‐alpha activity. (A) Scalp topography of *t* values from cluster‐based permutations contrasting baseline‐corrected high‐alpha (11–15 Hz) activity between the control group and the migraine group at the sensor level (time window of interest: 0.9–1.6 s post‐cue onset; baseline window: −0.6 to −0.2 pre‐cue onset). (B) Time courses of baseline‐corrected high‐alpha power at occipital sensors (highlighted with green circles in the topographic map) for the control and migraine groups. (C) Distributions of *t* values, masked at corrected *p* < 0.050, from cluster‐based permutation tests (two‐tailed test, cluster formation threshold at *p* = 0.050) contrasting baseline‐corrected high‐alpha activity during the time window of interest between the control group and the migraine group at the source‐level (positive values indicate a stronger activity in the migraine group). [Color figure can be viewed at wileyonlinelibrary.com]

At the sensor‐level, cluster‐based permutation analysis indicated that patients with migraine presented reduced high‐alpha (11–15 Hz) power in anticipation of the auditory target and during target processing compared to control participants. This corresponded to a significant cluster over occipital sensors, ranging from 900 to 1600 ms (*p* = 0.048). Based on this result, high‐alpha activity during the 900–1600 ms time window was reconstructed to localize the group effect. Source analysis indicated that the larger increase of alpha power observed among control participants emerged from a cluster including bilateral occipital cortices and the right dorso‐frontal cortex (*p* = 0.043) (Figure 3).

No significant group difference was found in the low‐alpha band at the sensor‐level (*p* > 0.999). To confirm this null result, low‐alpha activity in anticipation of the target (700 to 1100 ms time window) was reconstructed. Source analysis showed no significant difference in low‐alpha power between the migraine and control groups (*p* > 0.31).

### Distractor‐elicited gamma‐band activity

**FIGURE 4:**
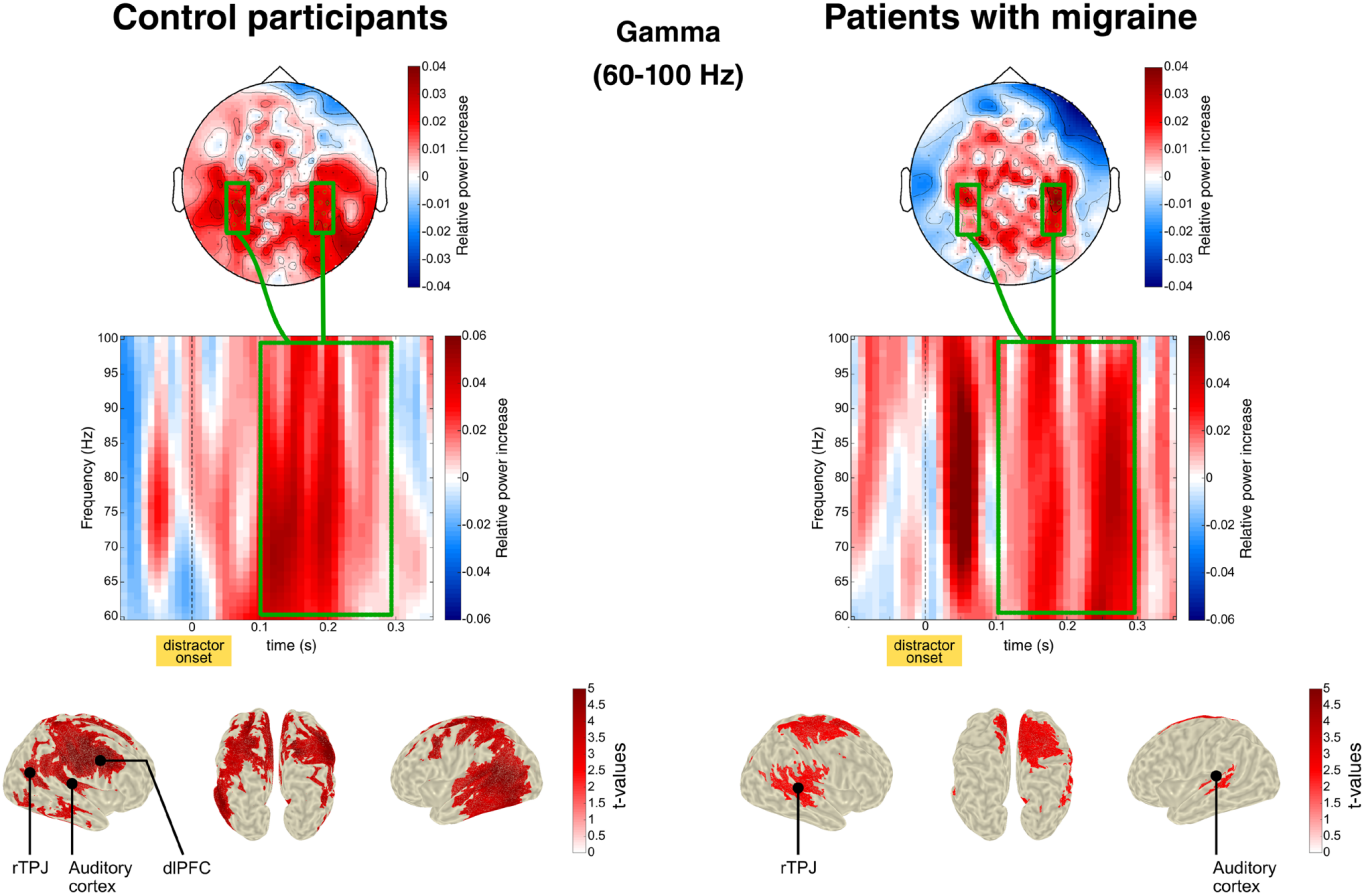
Distractor‐related gamma activity in each group. (Top) Scalp topographies of the baseline‐corrected gamma power (frequency of interest: 60–100 Hz; time window of interest: 0.1 to 0.3 s post‐distractor onset). (Middle) Time‐frequency visualization of baseline‐corrected gamma power measured at the temporo‐parietal sensors highlighted with green circles in the topographic map. (Bottom) Distributions of *t* values, masked at corrected *p* < 0.05, from cluster‐based permutation tests (one‐tailed tests, cluster formation threshold at *p* = 0.005 for the control group, *p* = 0.010 for the migraine group) contrasting real and surrogate distractor gamma activity during the time‐window of interest at the source level. rTPJ, right temporo‐parietal junction; dlPFC: dorsolateral prefrontal cortex. [Color figure can be viewed at wileyonlinelibrary.com]

The distracting sound elicited in both groups an increase of gamma power (60–100 Hz) between 100 and 300 ms at the sensor‐level. This increase of gamma power was visible over a large number of sensors but was maximal over a left and a right cluster of temporo‐parietal sensors. In the control group, source analysis indicated that this increase of gamma‐band power corresponded to a significant cluster including the bilateral temporo‐parietal junctions, both auditory cortices, and the right dorso‐lateral prefrontal cortex (*p* < 0.001). In the migraine group, source analysis indicated that the increase of gamma‐band power corresponded to four significant clusters including the rTPJ, the right sensorimotor cortex, and both auditory cortices (*p* < 0.010 for all four clusters) (Figure 4).

No significant group difference in gamma power between 100 and 300 ms was found at the sensor, nor at the source level (*p* > 0.999).

## DISCUSSION

Migraine has been associated with attentional abnormalities, as evidenced by subjective reports,^14^, ^15^, ^64^ neuropsychological testings,^65^, ^66^, ^67^ EEG, and event‐related potential studies.^50^ However, the functional role of brain oscillations in the disruption of attention in migraine remains poorly understood. In the present study, capitalizing on previous studies using the Competitive Attention Task, we investigated alpha activity during top‐down anticipatory processes and gamma activity during bottom‐up processing of distracting auditory stimuli in patients with migraine without aura during the interictal phase. Compared to control participants, patients with migraine displayed reduced top‐down inhibitory processes, as revealed by a reduced alpha synchronization (i.e., power increase) in visual areas during anticipation of an auditory target. In contrast, top‐down facilitatory processes and bottom‐up processes, as reflected by alpha desynchronization and gamma‐band activity, respectively, were preserved in the migraine group.

### Top‐down anticipatory processes in migraine

There is growing consensus that alpha rhythms reflect active functional inhibitory processes through the modulation of neuronal excitability.^34^, ^35^ During attention tasks, attending to the location, feature, or timing of a stimulus results in a decrease of alpha power in sensory areas relevant for its processing, and in an increase of alpha power in irrelevant sensory areas. These alpha modulations have been described in both the visual^68^, ^69^, ^70^, ^71^, ^72^, ^73^ and the auditory^38^, ^74^, ^75^, ^76^ modalities. During multisensory paradigms such as the one used here, alpha power has been reported to increase in brain areas processing the unattended sensory modality and to decrease in sensory relevant areas.^77^, ^78^, ^79^, ^80^ It has been specifically observed in a previous study in young healthy adults that the anticipation of an auditory target is associated with an increase of high‐alpha (11–15 Hz) power in the task‐irrelevant occipital cortex concomitant to a decrease of low‐alpha (7–11 Hz) power in the task‐relevant auditory cortex, reflecting distinct inhibitory and facilitatory attentional mechanisms,^36^ a finding confirmed in a different paradigm.^81^ Moreover, with the Competitive Attention Task used in our study, inhibition of the visual pathway was found functionally relevant, because behavioral performance positively correlated with the increase of (high) alpha power in the occipital cortex.^36^

In the present study, we confirm in the control group that the anticipation of an auditory target leads to (1) a decrease of low‐alpha power in the auditory cortex, interpreted as a facilitation of auditory processing; and (2) an increase of high‐alpha power in the occipital cortex, interpreted as an inhibition of visual processing. In contrast, patients with migraine showed a reduced and shorter‐lasting increase in alpha power increase in the occipital cortex in anticipation of the auditory target and during target processing. Patients with migraine appear to be less efficient at suppressing the task‐irrelevant visual pathways, which suggests that migraine is associated with deficient top‐down inhibitory processes, in keeping with inhibitory deficits reported in other cognitive domain (e.g., inhibition of irrelevant information in Stroop paradigms,^82^ response inhibition^83^).

Occipital cortical hyperexcitability has been proposed to be a feature of the migraine brain.^84^ For instance, patients with migraine exhibit highly synchronized steady‐state activity in the alpha band during flickering stimulation,^5^, ^13^, ^85^ suggesting impaired suppression of repetitive visual stimulation. Importantly, despite the above neurophysiological alterations, no group differences were observed in behavioral performance in either accuracy or reaction times in the absence of distractor. This dysfunction may be compensated by an enhanced engagement of top‐down attentional processes, as reflected in event‐related fields in the same patient group.^18^ Such a compensatory mechanism would be sufficient to maintain performance in controlled experimental context of a laboratory task, but may come at a cognitive cost in everyday environments, possibly contributing to sensory hypersensitivity commonly reported in migraine. Therefore, patients with migraine would use a different cognitive strategy to reach similar performance than control participants. Using the same attention task, we observed different brain responses (lower occipital alpha and motor mu power) but similar performance in the absence of a distractor in younger and older adults,^49^ suggesting again that distinct cognitive strategies (attentional in younger and motor in older) can result in similar performance.

In addition, patients with migraine presented a decreased high‐alpha power in dorsal prefrontal areas compared to control participants, suggesting an increased cortical activation. The dorsal prefrontal cortex has been consistently associated with goal‐oriented, top‐down attentional processes,^86^, ^87^ and as part of the dorsal attention network, it has been proposed to coordinate alpha power modulation in sensory cortices.^74^, ^75^, ^88^, ^89^, ^90^, ^91^ Overactivation of the dorsal prefrontal cortex in migraine could reflect an inefficient top‐down regulation of sensory areas, potentially contributing to the visual cortical hyperexcitability.

By contrast, no significant group difference was observed for the low‐alpha decrease in the auditory cortex. One could have expected an inadequate alpha decrease in the auditory cortex in anticipation of an auditory target, but our result suggests that top‐down facilitatory processes remain intact. In previous works investigating top‐down attention in migraine, patients displayed different patterns regarding facilitatory and inhibitory attentional mechanisms.^92^ On the one hand, patients with migraine appear to present a better‐than‐normal ability to facilitate in a top‐down fashion the processing of visual^92^ or auditory target stimuli.^18^ These group differences emerged during target processing, not during the anticipatory period, suggesting mainly reactive facilitatory processes, rather than proactive anticipatory processes. On the other hand, migraine appears to be associated with an impairment of visual noise suppression,^93^ and with a decreased ability to suppress unattended inputs in the periphery.^20^, ^92^ Overall, migraine seems to be characterized by a normal or even increased facilitation of processing of relevant inputs, but importantly, also by a deficient ability to proactively suppress irrelevant information. Overall, the allocation of attention across sensory modalities might not be optimal in patients with migraine. In keeping with this hypothesis, the functional connectivity between visual and other sensory (notably auditory) cortices has been observed to be increased in migraine with resting‐state functional magnetic resonance imaging.^94^ However, whether this impaired top‐down inhibition in migraine is specific of the visual system or extends to other modalities remains an open question.

### Bottom‐up attentional processes in migraine

Gamma activity has been associated with attention mechanisms. During attention tasks, gamma activity behaves opposite to alpha rhythms, because gamma power is enhanced in task‐relevant areas.^46^, ^95^ Gamma activity is proposed to reflect feedforward, bottom‐up processes, contrary to alpha rhythms, which are more closely associated with feedback, top‐down mechanisms.^77^, ^96^

As previously observed,^43^ we found that distracting sounds trigger an increase of gamma power within the VAN and auditory cortices in both healthy and migraine participants. This distractor‐induced gamma burst is interpreted as being the physiological manifestation of attention capture by distracting sounds, because the VAN is considered to underlie stimulus‐driven, bottom‐up attention mechanisms.^31^, ^87^, ^97^ In the present study, contrary to our a priori hypotheses, patients with migraine do not appear to present an enhanced recruitment of the VAN in response to a salient, unexpected sound, as assessed with the study of gamma‐band activity.

In a prior analysis of the same data set,^18^ patients with migraine displayed increased event‐related responses to distracting sounds, namely the orienting response of the N1 (negativity at 100ms) and the reorienting negativity (RON), and presented during the RON an increased recruitment of the rTPJ. The rTPJ is a crucial node of the VAN linked to bottom‐up attention orienting, and is also strongly involved in attention shifting.^31^ The absence of group difference in the distractor‐elicited gamma activity in the VAN might seem surprising in light of previous results.

However, distraction is not limited to the sole attention capture and is generally conceptualized as a three‐stage process.^98^, ^99^ First, a change detection mechanism, reflected by the orienting component of the N1 is elicited by sudden, infrequent sounds.^100^, ^101^, ^102^ This detection mechanism would trigger an attention capture by the unexpected sound, through the activation of the VAN^43^, ^103^ and reflected by the P3a (first positivity at 300 ms).^32^ Finally, participants would reorient their attention back to the task at hand, as reflected by the RON.^104^ These three steps are not necessarily tightly coupled and their respective electrophysiological markers may vary independently.^99^, ^105^ In migraine, the second stage, attention capture, does not seem to be negatively impacted, as evidenced by the typical distractor‐elicited P3a^18^ and gamma activity in the VAN (present results). However, the initial change detection mechanism could be more easily triggered in migraine, as evidenced by the heightened N1 (for converging evidence in passive auditory listening^16^, ^19^), without necessarily leading to an exacerbated attention capture. The heightened RON and increased recruitment of the rTPJ following distractors could then be seen as a compensatory top‐down mechanism that facilitates the reallocation of attention back to the task, allowing patients with migraine to better recover from the heightened change detection. On the one hand, a more prominent change detection mechanism (reflected by a larger N1) could explain why patients with migraine frequently self‐report being more easily disturbed by salient sounds.^14^ On the other hand, the presence of a typical bottom‐up attention capture (as indexed by typical distractor‐elicited P3a^18^ and gamma activity in the VAN), associated with a stronger top‐down reorientation process (evidenced by increased RON and rTPJ activity following distractors), could account for the absence of increased behavioral costs from distractors during the present task. Using an analogous attention task, we observed different brain responses but similar performance in high and low dream recallers,^106^ suggesting again that distinct cognitive strategies can result in similar performance.

### Clinical perspectives

In an environment filled with multiple sources of sensory stimulation, top‐down selective attention enables individuals to focus on relevant stimuli while ignoring irrelevant ones, whereas bottom‐up attention allows individuals to remain aware of unattended but potentially meaningful events. Based on the present results, migraine seems to be associated with a deficient recruitment of top‐down inhibitory mechanisms; patients with migraine appear to be less able to inhibit activity in visual areas when these regions are not task‐relevant. In contrast, there is no evidence that top‐down facilitatory mechanisms are impaired in migraine, in agreement with the previous literature on the subject. Similarly, the recruitment of the ventral attention network was normal in patients with migraine, suggesting that they may somehow manage to maintain normal bottom‐up attention capture to unexpected sounds, despite a general state of hyperresponsiveness.

The apparent inability of patients with migraine to suppress irrelevant information may be part of the neurophysiological underpinning of their complaints of attention difficulties and increased distractibility in their daily life.^14^, ^15^, ^50^, ^64^ A better understanding of the attentional alterations associated with migraine might inform therapeutical strategies to improve patients' daily life and possibly reduce attack frequency and fatigue by adopting cognitive strategies adapted to their interictal brain functioning.

Based on the present results, patients with migraine would not have major problems focusing on their work but would fail to effectively suppress irrelevant sensory inputs, notably in the visual modality. This finding calls for an exploration of attentional processing in more realistic contexts in migraine, with concurrent dynamic stimuli in all sensory modalities. Clinical training specifically focusing on distraction and noise suppression may be useful for symptom management. Finally, deficient top‐down inhibitory mechanisms may play a role in multimodal hypersensitivity during both the headache phase and the pain‐free period.^8^, ^10^, ^11^, ^14^ Further research is needed to establish a clearer relationship between sensory complaints and the disruption of the attention processing of sensory stimuli in migraine.

### Limitations of the study

Several limitations necessitate mentioning. In the present cross‐sectional study, data from only 19 patients experiencing migraine without aura and 19 matched controls were analyzed. The limited number of participants increases the risk of type II error (i.e., false negative); hence, the null results for behavioral data should be taken with caution. It remains to be tested in larger‐scale studies with more diverse profiles of patients whether the pattern of attention atypicalities observed here generalizes to migraine with aura, chronic migraine, and in preventive medication users, and also, to make the link between subjectively experienced difficulties and brain markers of attention. It is also worth noting that the cross‐sectional design precludes any conclusion as to when and how attentional deficits arise in migraine pathophysiology; for example, the decrease in visual alpha oscillations in patients with migraine could be a causal deficit but also an adaptive change.

## CONCLUSION

The present study is the first exploration of attention processes with MEG recordings in migraine without aura. The results reveal that patients with migraine have a reduced inhibition of task‐irrelevant visual processing during the performance of an auditory task, as indexed by alpha oscillations in visual cortices. Future studies could analyze more finely the relationships between sensory hypersensitivity and attention atypicalities in migraine, with and without aura, in more realistic contexts.

## AUTHOR CONTRIBUTIONS

**Rémy Masson:** Conceptualization; writing – original draft; visualization; formal analysis; investigation. **Alma ElShafei:** Formal analysis; investigation; writing – review and editing. **Geneviève Demarquay:** Conceptualization; writing – review and editing. **Lesly Fornoni:** Investigation; writing – review and editing; formal analysis. **Yohana Lévêque:** Writing – review and editing; investigation; conceptualization. **Anne Caclin:** Conceptualization; funding acquisition; supervision; writing – review and editing. **Aurélie Bidet‐Caulet:** Conceptualization; funding acquisition; supervision; writing – review and editing.

## FUNDING INFORMATION

This work was supported by the French National Research Agency grant ANR‐14‐CE30‐0001‐01 (to Aurélie Bidet‐Caulet and Anne Caclin). This work was performed within the framework of the LABEX CORTEX (ANR‐11‐LABX‐0042) and the LABEX CeLyA (ANR‐10‐LABX‐0060) of the Université de Lyon, within the program Investissements d'Avenir (ANR‐16‐IDEX‐0005) operated by the French National Research Agency.

## CONFLICT OF INTEREST STATEMENT

**Rémy Masson**, **Alma ElShafei**, **Geneviève Demarquay**, **Lesly Fornoni**, **Yohana Lévêque**, **Anne Caclin**, and **Aurélie Bidet‐Caulet** declare no conflicts of interest.
